# Formulation optimization of functional wheat bread with low glycemic index from technological and nutritional perspective

**DOI:** 10.1002/fsn3.3060

**Published:** 2022-09-15

**Authors:** Neda Mollakhalili‐meybodi, Mohammad Hassan Ehrampoush, Bahador Hajimohammadi, Mohammad Hassan Mosaddegh

**Affiliations:** ^1^ Department of Food Science and Technology School of Public Health Shahid Sadoughi University of Medical Sciences Yazd Iran; ^2^ Research Center for Food Hygiene and Safety Shahid Sadoughi University of Medical Sciences Yazd Iran; ^3^ Environmental Science and Technology Research Center Department of Environmental Health Engineering School of Public Health Shahid Sadoughi University of Medical Sciences Yazd Iran; ^4^ Department of Food Hygiene and Safety School of Public Health Shahid Sadoughi University of Medical Sciences Yazd Iran; ^5^ Department of Pharmacology School of Pharmacy Shahid Sadoughi University of Medical Sciences Yazd Iran

**Keywords:** inulin, nutritional, reconstitution, supplementation, technological, wheat bread

## Abstract

Inclusion of prebiotic compounds as indigestible dietary fiber in wheat bread has grown rapidly considering the increased public awareness about their impact on health. However, through their incorporation, the technological characteristics may adversely be influenced by gluten dilution impacts. This study was done to evaluate the impacts of long chain, native and short chain inulin (L‐, N‐, and S‐type inulin, respectively) at 8%, 10%, 12%, 14%, and 16% w/w as Inulin Reconstituted Wheat Flour (IRWF) with similar gluten: carbohydrate ratio of wheat flour (at 10%, 12.5%, 15%, 17.5%, 20% w/w) on technological and nutritional value of wheat bread. Results indicated that despite no gluten dilution induced by IRWF supplementation, technological characteristics were adversely influenced especially at higher substitution level of L‐type‐containing formulations which is attributed to their higher water absorption index (WAI). Reversely, the nutritional value was positively influenced in which the lowest hydrolysis index (26.64%); predicted Glycemic Index (51.93%) and fructan loss content (25.42%) were found at L‐type inulin‐containing IRWF at the highest substitution level (20% w/w). As the nutritional value of wheat bread as staple foodstuff is important, optimizing the bread‐making process to decrease all reverse impacts induced by L‐inulin‐type inclusion seems to be required.

## Graphical abstract

Impact of inulin incorporation as a strategy of wheat bread fortification is investigated. Inulin reconstituted wheat flour is used to postpone its gluten dilution impact. Technological characteristics of wheat bread are determined by water absorption index of formulations. Nutritional characteristics of fortified wheat bread are influenced by polymerization degree of inulin.

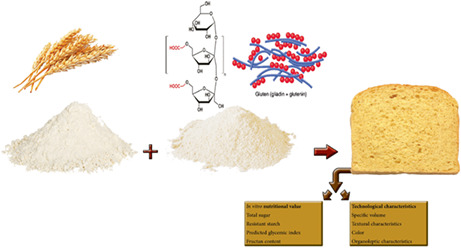

## 1 INTRODUCTION

Bread is recognized as one of the main foods consumed globally. Regarding World Health Organization (WHO) recommendation, the per capita consumption of bread is estimated at about 250 g per day (Serrem, 2011). However, it is consumed at a higher ratio especially in cereal‐based diet societies (developing countries). In Iran, its consumption rate is estimated at about 320 g per day.

Bread is prepared in different shapes, sizes, and textures through the world using the combination of flour, water, salt, and yeast (sourdough) (Bansal & Kapoor, 2015). Wheat flour is preferred to be used in bread‐making process regarding its distinctive viscoelastic characteristic to provide gluten network to restore gases produced throughout the bread‐making process. Despite the nutritional and technological desirability of wheat bread, its high glycemic index (GI) will be a threat for health, especially in people who suffer from type II diabetes (Borczak et al., 2018).

GI is calculated by the ratio of area under curve (AUC) in response to a test and reference food consumed by one on different days and standard situations. AUC is an indicator of blood glucose response (Reka et al., 2017). In this regard, foods with GI lower than 55, 55–69, and above 70 are known as low, middle, and high GI foods, respectively (Rahelić et al., 2011). The GI of white wheat bread is 100 (Schneider et al., 2015). Low GI food consumption is preferred by those who are trying to lower their blood glucose level, for example, diabetic patients.

Carbohydrate fractions of food supplies like cereal differ in size, shape, chain length, amylose: amylopectin ratio, and branching degree (Gourineni et al., 2017; Zhang & Hamaker, 2009). The postprandial glucose response of this fraction is determined by its digestibility ratio. In this regard, starch is classified as rapidly and slowly digestible starch abbreviated as RDS and SDS, respectively, and resistant starch (RS) (Chung et al., 2009). High incorporation of RS and SDS are claimed to have low GI (Bharath Kumar & Prabhasankar, 2014).

Regarding the high GI of white wheat bread as a staple foodstuff, any attempt to reduce the portion of carbohydrate and its digestibility is appreciated especially for people who suffer from obesity, diabetes, and insulin resistance. Using protein, fat and fiber are considered as the main strategies to reduce the GI of foods rich in carbohydrates like wheat bread (Wee & Henry, 2020). Considering the importance of carbohydrates in the technological characteristics determination of wheat bread, using dietary fiber is recommended to be useful in the formulation of low GI wheat bread.

Prebiotics as indigestible carbohydrates act as bifunctional agents. In other words, their incorporation in food formulations, however decreases their postprandial glucose response, and enhances the growth/activity of probiotic bacteria in the gastrointestinal tract (Carlson et al., 2017; Mohebbi et al., 2018). Among different compounds known as prebiotic, Inulin as a polymeric compound of fructose with a polymerization degree of 2–60, joined by β1‐2 linkages is one of the most commonly used compounds in food formulations (Shoaib et al., 2016). Inulin is categorized considering its degree of polymerization (DP) as short chain (S) (DP ≤10), long chain (L) (DP ≥23), and native inulin (N) (DP: 2–60).

Inulin is found naturally in some plants, microorganisms or produced enzymatically from sucrose syrup using fructosyl transferases (Mollakhalili‐Meybodi et al., 2021). Regular intake of inulin as a prebiotic compound can stimulate the growth of bifidobacteria, produce short‐chain fatty acids (SCFAs) and lower the intestinal pH, thus reducing the activity of pathogenic bacteria, cholesterol absorption, and colon cancer risk, alongside the reduction of GI in its incorporated food supply (Sirbu & Arghire, 2017). However, keeping the intact structure of inulin through food formulation and processing is prerequisite to be efficient as prebiotic; the technological and organoleptic characteristics of wheat bread should not be also negatively affected to guarantee its regular intake by the consumers. As the dietary fiber incorporation can adversely impact the technological characteristics of wheat bread through gluten dilution impact, reconstitution technique was used in this study. In other words, the quantities of starch and gluten were determined in wheat flour and used at the same ratio in Inulin Reconstituted Wheat Flour (IRWF) (Arya et al., 2016).

Considering the importance of polymerization degree of inulin in determination of its technological characteristics efficiency, digestibility and processing susceptibility, the aim of this study was to assess the impacts of inulin DP and IRWF incorporation level on technological and in vitro nutritional value of wheat bread.

## 2 MATERIAL AND METHODS

### 2.1 Materials

Inulin (derived from chicory and at S, N, and L types) was provided by SENSUS (Netherland). All flours had at least inulin content of more than 99.5% w/w. Wheat flour and vital gluten protein were purchased from Roshan and Hedia companies (Yazd, Iran), respectively. Wheat flour contained moisture, ash, and protein in quantities of about 13.95%, 0.23%, and 10.93% w/w, respectively. Yeast was obtained by Khuzestan Yeast Company (Dezful, Iran) and the other constituents (salt, sugar, and canola oil) were provided from the local supermarket. All chemicals including sodium hydroxide, potassium hydroxide, hydrochloric acid, sodium borohydride, parahydroxybenzoic acid, sodium acetate, acetic acid, maleic acid, tris‐maleate buffer (pH 6.9), D‐fructose, α‐amylase, sucrase, and sodium carbonate were obtained by Merck Company (Germany). Pancreatic ɑ amylase, sucrase, β‐amylase, pepsin, pullulanase, inulinase, invertase, α‐glucosidase, and α‐cellulose were also provided by Sigma‐Aldrich company (America).

### 2.2 Method

This study with the aim of studying the potential of Inulin incorporation in reconstitution of wheat bread from technological characteristics and nutritional value perspective was set in full factorial experimental design. So, the impact of inulin DP (S, L, and N) and substitution level of inulin reconstituted wheat flour (IRWF) was investigated as demonstrated in Table 1. The proportion of gluten and inulin was similar to the gluten: starch ratio of basis wheat flour which is 15.33% w/w and 61.32% w/w, respectively.

**TABLE 1:** Formulations of the present study on the basis of inulin polymerization degree and its incorporation level

Number	Independent variables			Trial
	Polymerization degree of Inulin	Inulin incorporation level (% w/w)	Gluten incorporation level (% w/w)	
1	S	8	2	F1
2	S	10	2.5	F2
3	S	12	3	F3
4	S	14	3.5	F4
5	S	16	4	F5
6	L	8	2	F6
7	L	10	2.5	F7
8	L	12	3	F8
9	L	14	3.5	F9
10	L	16	4	F10
11	N	8	2	F11
12	N	10	2.5	F12
13	N	12	3	F13
14	N	14	3.5	F14
15	N	16	4	F15
16	‐	‐	‐	F16

*Note*: S: (short‐chain inulin with DP ≤10); N: (native inulin with DP range of 2–60); L: (long chain inulin with DP ≥23). F16 is the control sample.

### 2.3 Farinograph

Brabender farinograph (Brabender, Germany) was used to determine the quantity of water to reach the consistency of 500 BU according to the AACC Method 54‐21A. In this regard, the gluten and inulin were mixed well with wheat flour into the 300 g mixing bowl of farinograph which was operated at 30 ± 0.2°C. Farinograph curves were used for determination of the water absorption (percentage of water needed to provide a dough consistency of 500 BU), dough development time (time to reach a consistency of 500 BU), stability time (time in which the dough consistency is over 500 BU) and falling quality number were obtained.

### 2.4 Preparation of bread

Bread preparation on the basis of formulations represented in Table 1 was done as follows with all ingredients added on flour dry basis. So, active dry yeast, sugar, salt, and canola oil at 2.2%, 0.5%, 1% and 3% w/w were mixed. The water inclusion level was on the basis of farinograph data calculation. The mixture was then stored inside the fermentation cabinet for 4 h at 29 ± 0.5°C. The baking process was done in a convection oven at 220 ± 10°C. Determination of proper baking time was according to feasibility of dough separation from the pan.

### 2.5 Physical characterization of bread

#### 2.5.1 Specific volume

Rape Seed displacement method was used for volume determination of loaves according to AACC 109 method 10–05. After volume and weight determination, specific volume was determined by their ratio as follows:

(1) $$\text{Specific loaf volume}=\frac{\text{volume}\;\left({\mathrm{cm}}^{3}\right)}{\text{weight}\;\left(g\right)}$$

#### 2.5.2 Textural parameters

Instrumental texture profile analysis of breads was done and followed using texture profile analyzer (TA 20., KOOPA). Regarding, a 5 kg loading cell and cylinder probe (diameter of 43 mm), bread crumb with dimension of 20 × 20 × 25 mm was pressed up to its half height at speed ratio of 1 mm/s. The analysis was done in five replicates at room temperature. Texture profile analysis curves were assessed for hardness, springiness, and chewiness determination (Mohammadi et al., 2021).

### 2.6 Crust and crumb color estimation

The Hunter Lab instrument was used for color determination of bread (both crust and crumb). Results are expressed as L* (brightness/darkness), a* (redness/greenness), and b* (yellowness/blueness) indices (Shiri et al., 2021).

### 2.7 Sensory evaluation

Bread samples were sensory evaluated by nine‐point hedonic scale. In this regard, 1, 2, 3, 4, 5, 6, 7, 8, and 9 were indicators of extremely unpleasant, very much unpleasant, moderately unpleasant, slightly unpleasant, generally acceptable, slightly pleasant, moderately pleasant, very much pleasant and extremely pleasant bread samples, respectively. Color, flavor, texture, and overall acceptability were assessed as qualitative parameters by 30 semi‐trained panelists (male: female ratio of panelists was 50:50 with the range of 18–58 years old). So, encoded bread samples were served randomly. The panelist's healthiness (with no allergy to gluten protein) and eagerness to be participated were also considered (Menon et al., 2015).

### 2.8 Nutritional value

#### 2.8.1 Total sugar (TS)

Total sugar content was quantified as follow (Albalasmeh et al., 2013). Regarding, 10 μl of sugar extract (by adding 1 ml of 0.005 M acetic acid to 50 mg bread sample, vortexing for 10 min and centrifuging at 12000 × *g* for 10 min [Figueroa & Khan, 1991]) was transferred to 5% aqueous solution of phenol (300 μl). After incubation for 5 min, 1.8 ml of concentrated sulfuric acid (18.4 M) was added. They were vortexed for 30 s and incubated in a water bath at room temperature for 20 min. Then, absorption was read at 490 nm. Total sugar was calculated as mg glucose/g extract.

#### 2.8.2 Resistant starch (RS)

The RS was estimated on the basis of the method described by (Reshmi et al., 2017). The protein removal was done by incubation of 100 mg sample with pepsin solution (40,000 U/ml, 1 g/10 ml KCl‐HCl buffer) at 40°C for 60 min. Pancreatic α‐amylase solution (40 mg a‐ amylase: 200 U/ml) was used for 16 h at 37°C to hydrolyze starch. After washing with ethanol (99% v/v) and centrifuging, the supernatant pellets were digested with 2 M potassium hydroxide. Incubation of digested pellet and supernatant was done separately with amyloglucosidase (3300 U/ml) and the released glucose was measured by GODPOD kit and absorbance at 510 nm. The RS and Digestible starch (DS) were quantified as follows:

(2) $$\mathrm{RS}:\text{glucose}\;\left(\mathrm{mg}\right)\times 0.9$$

(3) DS:TS−RS

#### 2.8.3 Predicted glycemic index (pGI)

In vitro method was used to determine the pGI on the basis of the procedure proposed by (Reshmi et al., 2017). Regarding, 100 mg of ground bread sample was incubated with HCl–KCl buffer (10 ml and pH 1.5) and 200 μl pepsin solution (100 mg/ml HCl‐KCl buffer) for 1 h at 40°C. By adding 200 μl pancreatic α‐amylase solution (1.5 mg/10 ml phosphate buffer), the pH was increased to 7.8 and incubated for 45 min at 37°C. To stop the enzyme reaction, Na_2_CO_3_ solution was added at 70 μl and using trismaleate buffer at pH = 6.9, samples were diluted to 25 ml. Then, 5 ml of pancreatic α‐amylase solution (3 U/5 ml tris‐maleate buffer) was added and incubation was done at 37°C.

About 1 ml of samples were treated with 3 ml of 0.4 M sodium acetate buffer adjusted at pH equal to 4.75 and 60 μl amyloglucosidase (3300 U/ml) and incubated at 60°C for 45 min. Afterward, distilled water was used for the volume adjustment to 10 ml. The solution (0.1 ml) was transferred into glass test tubes for glucose quantification by GODPOD kit and absorbance at 510 nm. The hydrolysis index (HI) was estimated as the ratio of total released glucose of samples and standard glucose (Barine & Yorte, 2016). The pGI of the samples was calculated on the basis of the following equation (Nathakattur Saravanabavan et al., 2013);

(4) pGI=39.71+0.549HI

#### 2.8.4 Fructan content quantification

The quantification of inulin in bread formulations was done enzymatically. In this regard, about 1 g bread sample was dissolved in 80 ml water (80°C). The mixture was filtered and treated with 1 ml sucrase/amylase solution (sucrase (50 U), β‐amylase (500 U), pullulanase (100 U), and maltase (1000 U) in 22 ml sodium maleate buffer (pH 6.4) to remove sucrose, starch and reducing sugars). Afterward, alkaline borohydride (10 mg/ml sodium borohydride in 50 mM NaOH) was added and incubated at 40°C for 30 min and the excess was removed using acetic acid (100 mM) to reach the pH equal to 4.5. Quantification of fructan was done by fructanase (350 U/ml exo‐inulinase and 35 U/ml endo‐inulinase) for 20 min at 40°C. The fructan content (%) was estimated as:

(5) $$\text{Total fructan}\;\left(\%\right)=A\times F\times 5\times V\times 1.1/0.2\times 100/w\times 1/1000\times 162/180$$

where:

A: absorbance of 0.2 ml of the reactive solutions read against the reagent blank.

F: equal to 54.5, factor used to convert the absorbance value to μg fructose.

V: volume of used extractant.

M: weight of test portion.

### 2.9 Statistical analysis

Quantitative characteristics were described using mean and standard deviation. All examinations were done in triplicate. Data analysis was performed using two‐way anova and Kruskal–Wallis for parametric and non‐parametric tests, respectively, by spss V.21 software at a significance level of 0.05.

## 3 RESULT AND DISCUSSION

### 3.1 Effect of IRWF on farinograph parameters

The viscoelastic network formation of dough is influenced greatly by the other ingredients (Meybodi et al., 2019). Wheat flour supplementation with IRWF changes the dough mixing characteristics significantly as determined by farinograph. Flour quality is determined by Farinograph test according to dough physical resistance through mixing and/or kneading. Farinograph characteristics of wheat flour and those supplemented with IRWF, at 10%, 12.5%, 15%, 17.5%, and 20% w/w are shown in Table 2.

**TABLE 2:** Farinograph characteristics of wheat flour supplemented with inulin‐containing reconstituted wheat flour

Samples	Parameters			
	WA (g/100 g)	DDT (min)	ST (min)	FQN (−)
F1	52.12 ± 0.16^g^	2.23 ± 0.12^k^	9.19 ± 0.03 ^i^	77.12 ± 0.07^h^
F2	54.09 ± 0.07^f^	2.27 ± 0.05^k^	11.09 ± 0.07^h^	81.12 ± 0.10^g^
F3	55.74 ± 0.01^e^	3.01 ± 0.09^j^	12.11 ± 0.14^g^	99.65 ± 0.51^g^
F4	57.01 ± 0.23^d^	3.64 ± 0.10^i^	13.06 ± 0.01^f^	108.71 ± 0.21^f^
F5	58.13 ± 0.01^cd^	4.08 ± 0.17^h^	14.28 ± 0.08^e^	112.01 ± 0.62^e^
F6	55.65 ± 0.09^e^	3.14 ± 0.12^j^	13.17 ± 0.21^f^	118.35 ± 0.09^c^
F7	56.17 ± 0.17^d^	4.53 ± 0.05^g^	14.08 ± 0.03^e^	105.08 ± 0.07^f^
F8	58.38 ± 0.05^c^	5.17 ± 0.04^f^	15.01 ± 0.23^d^	115.10 ± 0.05^d^
F9	59.14 ± 0.14^c^	6.04 ± 0.06^e^	16.37 ± 0.04^c^	118.35 ± 0.14^c^
F10	60.09 ± 0.02^bc^	7.13 ± 0.25^cd^	17.01 ± 0.08^b^	120.03 ± 0.51^c^
F11	58.09 ± 0.05^d^	6.87 ± 0.09^d^	15.25 ± 0.09^d^	112.92 ± 0.32^e^
F12	58.97 ± 0.08^c^	7.93 ± 0.08^c^	16.48 ± 0.10^c^	115.89 ± 0.43^d^
F13	60.57 ± 0.05^bc^	9.03 ± 0.21^b^	17.28 ± 0.17^b^	118.16 ± 0.21^c^
F14	61.09 ± 0.51^b^	9.43 ± 0.17^a^	18.71 ± 0.21^a^	123.14 ± 0.41^b^
F15	63.24 ± 0.01^a^	10.05 ± 0.16^a^	19.23 ± 0.42^a^	125.07 ± 0.32^a^
F16	51.07 ± 0.23^g^	2.13 ± 0.04^k^	8.73 ± 0.19^i^	63.02 ± 0.16^i^

*Note*: Data are reported as average ± standard deviation. The values with the different lowercase letters in each column mean the significant difference (*p* < .05).

Abbreviations: DDT: dough development time; FQN: farinograph quality number; ST: stability time; WA: water absorption.

As demonstrated, wheat flour supplementation with IRWF influenced the WA content in an inulin DP‐dependent manner (*p* < .05). In other words, while the ability to absorb water is remained quite unaffected at formulation containing 8% w/w S‐type inulin‐containing IRWF (F1) (*p* > .05), it has been increased in the presence of 8% w/w L‐type inulin‐containing one (F10) compared to control (F16). The WA enhancement at formulations supplemented with IRWF‐containing long chain inulin is mainly attributed to their ability to provide a gel‐like structure potentially able to restore water as reported by (Mohammadi et al., 2021). Regardless of inulin DP, it is clear that supplementation level is also influential in WA quantity determination of wheat flour which is in the range of 2.05%–15.43%, 8.93%–17.66%, and 15.46%–23.53% w/w for S, N, and L inulin‐containing IRWF (at ascending ratio), respectively. However, this enhancement is more obvious at long‐chain inulin‐containing formulations. The WA enhancement via inclusion of different vegetable protein has also been previously stated which is attributed to their difference in WA capacity and consequently increased WA value in farinograph plot (Coţovanu & Mironeasa, 2022).

Dough development time (DDT) is also found to be influenced by IRWF supplementation. DDT is the required time for flour to be hydrated, developed, and provided consistent dough equal to 500 Brabender Unit (BU) consistencies. Results indicated significant (*p* < .05) enhancement of DDT at all IRWF‐containing formula depending on polymerization degree of inulin and IRWF supplementation level (*p* < .05) (Table 1). DDT enhancement induced by IRWF inclusion could be attributed to physicochemical characteristics difference in constituents of IRWF and wheat flour (Paraskevopoulou et al., 2010). Increased DDT (*p* < .05) found by increasing the DP of incorporated inulin in IRWF formulation, is also attributed to higher susceptibility of L‐type inulin to interact with gluten proteins and prevent their hydration and development and consequently lead to DDT enhancement (Graça et al., 2020).

Stability time (ST) as stability indicator of dough is significantly influenced by dough constituents. As depicted, the lowest and highest ST are found in F16 (control) and F15 (long chain inulin‐containing IRWF at 20% w/w) with contents equal to 8.73 and 19.23 min, respectively. In other words, wheat dough supplementation using IRWF enhanced the ST significantly on the basis of its supplementation level and polymerization degree of incorporated inulin. In this regard, it seems that dough samples with higher IRWF incorporation level had higher ST and consequently mixing resistance compared to the control sample. Previously, it has been stated that differently originated proteins in dough mixtures are not fully hydrated and behaved as dispersed particles which reinforce the gluten network and consequently increase their derived dough ST (Paraskevopoulou et al., 2010).

Considering results obtained in Table 2, the farinograph quality number (FQN) which is an indicator of gluten protein quality and quantity is enhanced by IRWF supplementation level and inulin DP. In other words, the highest FQN is found in F15 with about 98.46% enhancement in FQN compared to control sample. As the FQN is an indicator of mixing tolerance and dough strength, it is suggested that IRWF incorporation enhances the bread‐making performance of wheat dough (Whitney & Simsek, 2020).

### 3.2 Effects of IRWF on physicochemical characteristics

The impacts of IRWF supplementation on physicochemical characteristics of wheat breads have been investigated and represented in Table 3.

**TABLE 3:** Physicochemical characteristics of wheat breads supplemented with inulin‐containing reconstituted wheat flour

Samples	Parameters			
	SV (cm^3^/g)	Hardness (g)	Chewiness (mJ)	Springiness (mm)
F1	2.63 ± 0.06^a^	812.32 ± 2.23^L^	84.36 ± 0.07^j^	9.14 ± 0.07^a^
F2	2.58 ± 0.02^b^	1009.48 ± 0.17^k^	92.76 ± 0.01^i^	8.94 ± 0.02^ab^
F3	2.53 ± 0.07^bc^	1319.67 ± 2.15^j^	115.49 ± 0.05^h^	8.54 ± 0.06^b^
F4	2.45 ± 0.05^c^	1523.71 ± 3.08^i^	130.09 ± 0.03^g^	8.06 ± 0.01^d^
F5	2.43 ± 0.08^c^	1817.14 ± 1.18^g^	145.12 ± 0.08^e^	8.22 ± 0.05^c^
F6	2.01 ± 0.03^d^	1620.86 ± 0.05^h^	129.72 ± 1.00^g^	8.03 ± 0.09^d^
F7	1.90 ± 0.09^d^	1892.31 ± 11.08^g^	134.17 ± 0.09^f^	8.68 ± 0.01^b^
F8	1.72 ± 0.01^e^	2134.19 ± 10.01^f^	142.03 ± 1.16^e^	7.10 ± 0.08^f^
F9	1.68 ± 0.04^e^	2315.49 ± 4.12^e^	151.18 ± 1.02^d^	7.03 ± 0.05^f^
F10	1.43 ± 0.03^f^	2618.72 ± 9.60^d^	161.12 ± 1.09^bc^	6.12 ± 0.04^h^
F11	1.58 ± 0.08^e^	2198.02 ± 4.38^f^	135.83 ± 0.08^f^	7.60 ± 0.03^e^
F12	1.51 ± 0.04^ef^	2445.29 ± 0.12^e^	147.69 ± 1.35^e^	6.78 ± 0.05^g^
F13	1.49 ± 0.06^f^	2743.81 ± 5.12^c^	158.00 ± 0.02^c^	6.07 ± 0.01^h^
F14	1.45 ± 0.05^f^	3025.13 ± 3.19^b^	169.15 ± 1.13^b^	5.56 ± 0.05^i^
F15	1.37 ± 0.01^g^	3189.11 ± 8.01^a^	183.14 ± 1.81^a^	5.82 ± 0.08^i^
F16	2.65 ± 0.08^a^	756.09 ± 1.13^m^	70.06 ± 0.04^k^	9.03 ± 0.03^a^

*Note*: Data are reported as average ± standard deviation. The values with the different lowercase letter in each column mean the significant difference (*p* < .05).

As depicted, specific volume (SV) is significantly influenced by supplementation level and the type of incorporated inulin. In other words, despite no significant difference between F16 (Control sample) and F1 (containing S inulin type and supplementation level at 10% w/w) (*p* ≥ .05), about 48.30% w/w decreased is found in SV of F15 compared to control sample (*p* < .05) at. As SV is affected by dough strength and its prospective in restoring gases through bread‐making (Kou et al., 2019; Whitney & Simsek, 2020), this reduction in IRWF supplemented formulations may be induced by dough elasticity reduction provoked by their weakening impacts on gluten network. However, no gluten dilution is observed in all supplemented formulations, it seems more than early fixing of structure in L‐type inulin‐containing IRWF formulation induced by their higher water content leads to retardation in the development of gluten network (Sabanis & Tzia, 2009). In other words, higher affinity of L‐type inulin to bound water resulted in its competence with gluten and consequently reduce its development and ability to restore gases produced through fermentation process. This finding is in accordance with (de Erive et al., 2020).

The textual characteristics of IRWF‐supplemented‐wheat breads in terms of hardness, chewiness, and springiness are demonstrated in Table 3. As depicted, the highest and lowest hardness is found in F16 and F15 at 756.09 and 3189.11 g, respectively. Changes in chewiness characteristics are also in similar order of harness. In other words, the crumb hardness and chewiness have been increased by IRWF supplementation level and polymerization degree of incorporated inulin (*p* < .05). Considering the impact of inulin on crumb hardness, however, it has previously stated that its incorporation in wheat bread provides a coarse crumb structure with higher hardness (Frutos et al., 2008; Sardabi et al., 2021; Sirbu & Arghire, 2017), its adverse impact has been found at this study which is improved by increasing the inulin degree of polymerization. On the other hand, despite decreased hardness and increased SV via gluten protein inclusion in steamed bread (Qian et al., 2019), a reverse order has been found at this study which is supposed to be induced by more coarse crumb structure formed by formulations replaced by IRWF at high level, especially those containing long‐chain inulin as demonstrated by (Mohammadi et al., 2021). As a less elastic system is formed through IRWF supplementation, it is also declared by springiness data. Springiness as an indicator of deformation ratio of a deformed product to its un‐deformed state after removal of deforming force is directly dependent on hardness and chewiness. Generally, any variation in formulation will be corresponded in springiness and chewiness differentiation (Eriksson et al., 2014). Increasing the springiness value denote a well‐developed gluten network with more elasticity in crumb. As demonstrated in Table 3, IRWF incorporation reduced the springiness value depending on its incorporation level and polymerization degree of incorporated inulin. In other words, it has been decreased by increasing the IRWF incorporation level and increasing the inulin DP with springiness values equal to 9.03 and 5.82 for control and F16 samples, respectively. The decrease observed in springiness characteristics of IRWF‐incorporated wheat bread formulations containing L‐type inulin could be attributed to their lower strength and gas retention capability of gluten network. However, incomplete starch swelling through baking will also restrict the sponge‐like formation in bread crumb. This result is verified (de Erive et al., 2020; Londono et al., 2015).

### 3.3 Effects of IRWF on color parameters

Color characteristics are considered to be influential in consumer's acceptance determination of bread alongside its textural and physicochemical characteristics. Two mechanisms of Maillard and caramelization reactions are simultaneously evolved in color development of bakery products like wheat bread (Anil, 2007). The crust and crumb color determination of IRWF‐supplemented wheat bread has been conducted by CIE L*a*b* color scale as represented in Table 4. The crust and crumb color are generally developed by Maillard/caramelization reactions and inherent color characteristics of formulation, respectively (Dhen et al., 2018). Crumb color is also influenced by structure forming characteristics of formulation through bread‐making process (NithyaBalaSundari et al., 2020).

**TABLE 4:** Crust and crumb color analysis of wheat breads supplemented with inulin‐containing reconstituted wheat flour

Trial	Crust			Crumb		
	L*	a*	b*	L*	a*	b*
F1	77.28 ± 0.01^c^	9.13 ± 0.01^a^	24.15 ± 0.01^e^	89.17 ± 0.05^c^	9.17 ± 0.05^a^	15.23 ± 0.01^f^
F2	76.14 ± 0.07^cd^	9.95 ± 0.06^a^	23.92 ± 0.01^e^	85.13 ± 0.11^e^	9.23 ± 0.01^a^	15.94 ± 0.11^f^
F3	75.79 ± 0.02^d^	9.84 ± 0.04^a^	23.17 ± 0.05^f^	82.56 ± 0.14^f^	9.36 ± 0.07^a^	16.32 ± 0.07^e^
F4	73.13 ± 0.09^d^	10.13 ± 0.01^a^	25.14 ± 0.08^e^	80.00 ± 0.08^g^	9.63 ± 0.03^a^	18.01 ± 0.03^c^
F5	72.18 ± 0.11^e^	10.27 ± 0.05^a^	27.92 ± 0.03^bc^	79.12 ± 0.21^g^	9.59 ± 0.01^a^	17.59 ± 0.01^d^
F6	78.00 ± 0.21^bc^	9.15 ± 0.07^a^	23.14 ± 0.01^f^	91.15 ± 0.17^a^	8.21 ± 0.05^b^	17.21 ± 0.09^d^
F7	77.18 ± 0.08^c^	8.23 ± 0.01^b^	23.56 ± 0.10^f^	90.83 ± 0.06^a^	8.00 ± 0.08^b^	19.14 ± 0.02^b^
F8	76.21 ± 0.08^c^	7.16 ± 0.08^c^	24.14 ± 0.09^e^	90.17 ± 0.03^b^	7.93 ± 0.02^b^	18.91 ± 0.21^c^
F9	76.09 ± 0.05^cd^	9.11 ± 0.05^a^	26.11 ± 0.07^d^	91.51 ± 0.05^a^	6.82 ± 0.04^c^	19.18 ± 0.17^b^
F10	75.73 ± 0.17^d^	7.69 ± 0.02^c^	28.91 ± 0.02^a^	91.00 ± 0.19^a^	8.00 ± 0.01^b^	19.02 ± 0.02^b^
F11	79.13 ± 0.06^b^	6.15 ± 0.02^d^	26.21 ± 0.01^d^	83.12 ± 0.11^f^	7.21 ± 0.00^c^	17.00 ± 0.08^d^
F12	79.00 ± 0.05^b^	6.32 ± 0.05^d^	27.14 ± 0.02^c^	85.17 ± 0.28^e^	6.95 ± 0.03^c^	17.23 ± 0.04^d^
F13	78.94 ± 0.10^b^	4.18 ± 0.01^f^	28.00 ± 0.08^b^	87.99 ± 0.23^d^	7.63 ± 0.04^b^	18.62 ± 0.09^c^
F14	80.01 ± 0.01^b^	7.09 ± 0.07^c^	28.23 ± 0.06^b^	88.54 ± 0.04^d^	6.12 ± 0.01	18.14 ± 0.11^c^
F15	81.12 ± 0.13^ab^	7.38 ± 0.03^c^	29.11 ± 0.03^a^	89.15 ± 0.10^c^	7.79 ± 0.06^b^	20.34 ± 0.05^a^
F16	83.56 ± 0.11^a^	5.88 ± 0.05^e^	19.82 ± 0.01^g^	87.19 ± 0.07^d^	5.36 ± 0.05^d^	18.83 ± 0.08^c^

*Note*: Data are reported as average standard error. Values with different lowercase letters according to Tukey's test are significantly different in each column (*p* < .05).

As demonstrated in Table 4, IRWF‐supplemented bread had significantly (*p* < .05) lower L*, and higher a* and b* in crust compared to the control bread. Considering the polymerization degree of inulin, the highest variation is found in S‐type inulin samples. In other words, the lowest L* value is found in F5 at 72.18. The lightness reduction via low DP inulin incorporation has been previously stated by (Aguiar et al., 2021) which was supposed to be induced by their more susceptibility to be involved in Millard reaction. Decreased L* along with increased a* of wheat bread crust was previously related to increased Millard reaction ratio by (de Erive et al., 2020; Skendi et al., 2010). As the trend of changes in crust L* is inconsistent with a* and b* values, it seems that the yellow‐red shade of proteins in crust color determination is also influensive (de Erive et al., 2020).

As mentioned previously, the crumb color characteristics of bread samples are influenced by their formulation and structure‐forming characteristics (Kou et al., 2019). As demonstrated crumb color characteristics of wheat bread is influenced by its supplementation level and polymerization degree of incorporated inulin (*p* < .05). In other words, the highest and lowest L* are found in F15 (supplemented by 20% w/w L‐type inulin‐containing IRWF) and F5 (supplemented by 20% w/w S‐type inulin‐containing IRWF) with values at 79.12 and 89.15, respectively. It seems that higher susceptibility of S‐type inulin to be fermented by yeast leads to more release of carbon dioxide and air bubble expanding through bread‐making process which resulted in more light scattering and consequently decreased L* value. Increased L* in F15 is accompanied by the highest b* and harness values which are in accordance with (Matos & Rosell, 2013).

### 3.4 Sensory evaluation of IRWF supplemented wheat bread

**FIGURE 1:**
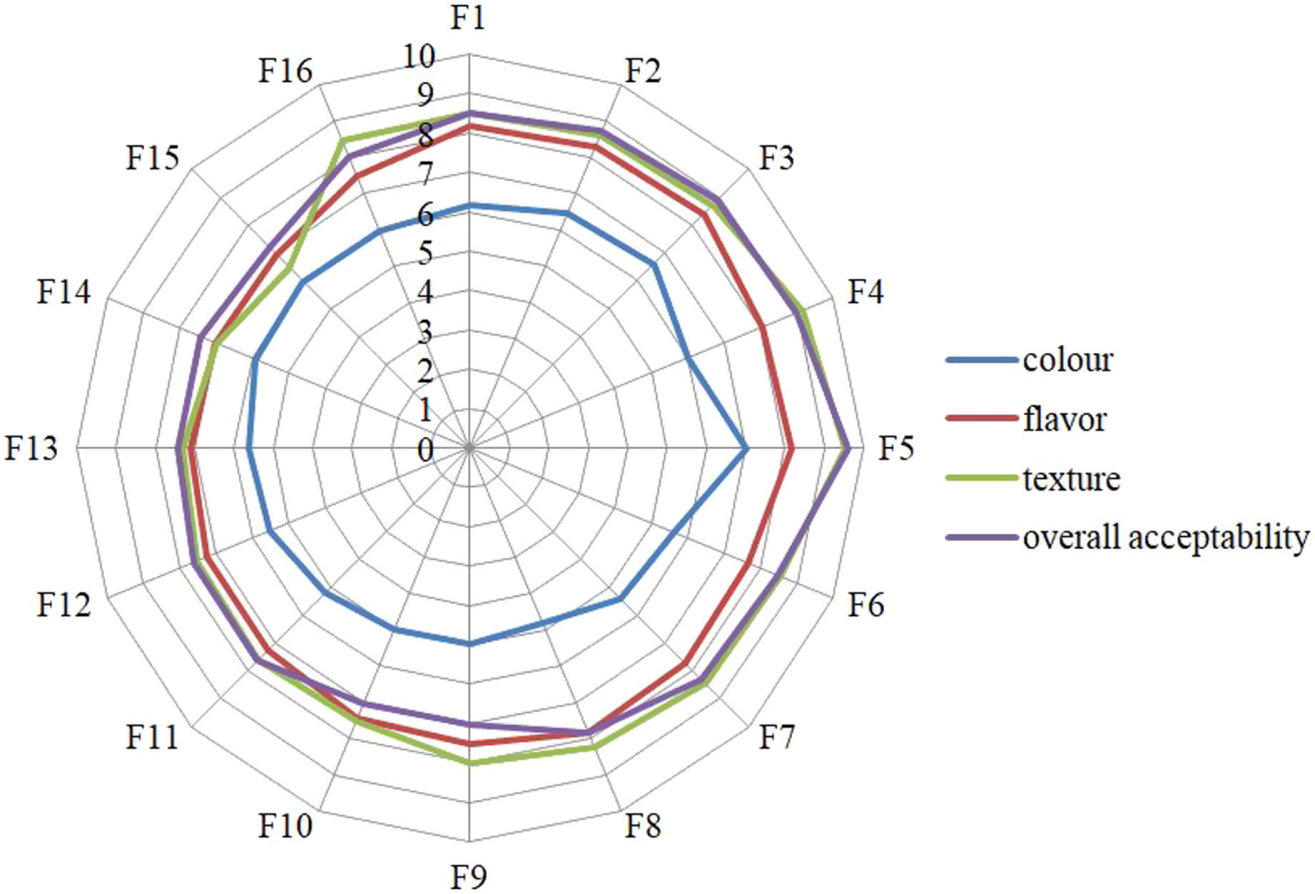
Radar plot for sensory evaluation of wheat breads supplemented with inulin‐containing reconstituted wheat flour

Considering the importance of the overall acceptability of new products to be introduced in market, sensory assessment of IRWF‐supplemented wheat bread was done and reported in Figure 1. Sensory characteristics were evaluated at four criteria of texture, flavor, color, and overall acceptability. As depicted, all samples are higher than mediate at all evaluated criteria. Generally, the highest score considering flavor, color, texture, and overall acceptability has been gained by F5 (containing S‐type inulin at IRWF supplemented level of 20% w/w). As the specific volume and crust color are the main indicators to meet the expectative of consumers (Di Monaco et al., 2015; Pasqualone et al., 2019), the optimization is found in F5.

Considering the flavor sensation, the highest score is found in samples containing S‐type inulin which can be attributed to higher metabolites of Millard reaction as described by (Mohammadi et al., 2021). The overall acceptability is well matched with texture characteristics (*r* = 0.87, *p* < .05).

### 3.5 Nutritional value

Nutritional value of wheat breads supplemented with IRWF as Total Sugar (S), Resistant Starch (RS), Digestible Starch (DS), Hydrolysis Index (HI), Predicted Glycemic Index (PGI), and fructan loss is presented in Table 5. As demonstrated, TS, RS and DS contents in the products are in the range of 64.13%–70.17%, 5.03%–10.19%, and 57.13%–68.09% on dry weight basis, respectively. Considering the control sample, incorporation of IRWF enhanced the RS and decreased the DS depending on its supplementation level and the polymerization degree of inulin incorporated in IRWF formulations. The decrease in TS seems to be more pronounced at formulations containing lower DP inulin. As the lower DP inulin is more susceptible to be hydrolyzed through bread making process, it will be more remarkably lost by Millard reaction through baking by Millard reaction (Morreale et al., 2019). However, this decrease will be reversely influenced by increasing the level of IRWF incorporation. The increased Millard reaction ratio through S‐type inulin incorporation is also obvious in crust color consideration. The highest RS value is found in F15 containing the highest IRWF supplementation level provided by higher DP inulin with amount at 10.19% which simultaneously has the lowest DS of 58.12%. Despite similar inulin incorporation level at different formulations, the difference in the rate of digestion seems to be induced by the differences in their nature and impacts on starch gelatinization. As the inulin gel formed at lower temperature, it prohibits the water accessibility for the amorphous region of starch and consequently restricts its gelatinization (Luo et al., 2017; Reshmi et al., 2017). Protein inclusion is also reported to reduce the starch susceptibility to enzymatic attack by decreasing the starch granule swelling and its gelatinization (Graça et al., 2021). In this regard, increased RS has been found in all formulations by increasing the level of IRWF.

**TABLE 5:** In vitro nutritional characteristics of wheat breads supplemented with inulin containing reconstituted wheat flour

Samples	Parameters					
	Total sugar	Resistant starch	HI	PGI	Digestible starch	Fructan loss
F1	68.01 ± 0.02^b^	5.32 ± 0.09^e^	42.59 ± 0.04^b^	63.09 ± 0.01^b^	63.13 ± 0.07^c^	43.01 ± 0.09^a^
F2	66.09 ± 0.05^d^	6.03 ± 0.12^d^	41.38 ± 0.05^c^	62.43 ± 0.08^c^	60.17 ± 0.04^d^	43.18 ± 0.05^a^
F3	64.13 ± 0.07^f^	6.32 ± 0.04^d^	40.49 ± 0.07^d^	61.94 ± 0.11^d^	58.91 ± 0.11^e^	43.09 ± 0.11^a^
F4	64.25 ± 0.03^f^	7.05 ± 0.06^c^	39.01 ± 0.09^e^	61.13 ± 0.06^d^	57.13 ± 0.09^f^	41.12 ± 0.06^b^
F5	65.37 ± 0.09^e^	7.35 ± 0.03^c^	37.26 ± 0.00^f^	60.17 ± 0.11^e^	68.09 ± 0.03^a^	41.00 ± 0.07^b^
F6	67.18 ± 0.01^c^	7.00 ± 0.07^c^	35.52 ± 0.12^g^	59.21 ± 0.09 ^f^	60.13 ± 0.16^d^	39.14 ± 0.01^c^
F7	67.03 ± 0.08^c^	8.61 ± 0.13^b^	31.69 ± 0.10^i^	57.11 ± 0.06^h^	59.21 ± 0.07^e^	38.54 ± 0.09^d^
F8	67.92 ± 0.04^b^	9.07 ± 0.08^b^	31.38 ± 0.04^i^	56.94 ± 0.04^h^	58.03 ± 0.12^f^	38.00 ± 0.03^d^
F9	67.89 ± 0.07^b^	9.21 ± 0.05^b^	31.51 ± 0.09^i^	57.01 ± 0.08^h^	58.11 ± 0.09^f^	37.19 ± 0.04^e^
F10	68.11 ± 0.04^b^	7.51 ± 0.02	30.01 ± 0.17^j^	56.19 ± 0.02^i^	61.03 ± 0.04^d^	36.71 ± 0.05^f^
F11	68.00 ± 0.02^b^	8.83 ± 0.03^b^	34.01 ± 0.05^h^	58.38 ± 0.07^g^	59.78 ± 0.06^e^	30.12 ± 0.09^g^
F12	68.02 ± 0.04^b^	9.05 ± 0.12^b^	29.76 ± 0.11^j^	56.05 ± 0.05^i^	59.81 ± 0.09^e^	29.18 ± 0.13^h^
F13	68.31 ± 0.03^b^	9.89 ± 0.07^a^	26.96 ± 0.01^k^	54.51 ± 0.01^j^	59.00 ± 0.12^e^	28.64 ± 0.04^hi^
F14	69.51 ± 0.04^a^	10.01 ± 0.04^a^	24.34 ± 0.08^L^	53.07 ± 0.09^k^	58.01 ± 0.05^f^	27.13 ± 0.07^i^
F15	69.87 ± 0.08^a^	10.19 ± 0.15^a^	22.26 ± 0.05^m^	51.93 ± 0.1^k^	58.12 ± 0.09^f^	25.42 ± 0.05^j^
F16	70.17 ± 0.07^a^	5.03 ± 0.02^e^	57.08 ± 0.03^a^	71.05 ± 0.05^a^	65.14 ± 0.07 ^b^	‐

*Note*: Data are reported as average standard error. Values with different lowercase letters according to Tukey's test are significantly different in each column (*p* < .05).

Hydrolysis indices (HI) which is calculated by comparison of glucose released from sample and standard glucose through the digestion process and its corresponding pGI is considerably influenced by the supplementation process. As depicted in Table 4, the highest and lowest HI is found at F16 and F15 with values at 57.08% and 22.6%, respectively, with simultaneously the highest and lowest pGI. In other words, increasing the polymerization degree of incorporated inulin significantly (*p* < .05) decreased the HI which are also decreased more sharply at formulations containing higher IRWF supplementation level. The decreased HI induced by increasing the level of IRWF and polymerization degree of incorporated inulin is attributed to decreased starch gelatinization ratio and its preventative impacts on hydrolyzing enzymes (Englyst et al., 2007; Garsetti et al., 2005; Gourineni et al., 2017). Considering the pGI, Foods with GI lower than 55, 56–69, and higher than 70 are categorized as low, middle, and high GI foods, respectively. The pGI of formulations prepared in this study ranged from 51.93 to 71.05 with all IRWF‐supplemented formulations as medium GI and F14 and F15 samples recognized as low GI ones (Reshmi et al., 2017). This observation is believed to be induced by increasing the level of RS and inulin and their inhibitory impacts on enzymes participated in post‐prandial glucose response. GI is a nutritional quality determinative parameter and diets with low GI and high RS are believed to be therapeutic in diabetic patients, reducing the insulin resistance and blood glucose adjustment, improving the lipid metabolism, and preventing the cardiovascular diseases (Chung et al., 2011; Reshmi et al., 2017).

Inulin is incorporated as a functional ingredient in this study through its prebiotic role. Consequently, its stability through bread making process is considerably important, as influensive prebiotics needs to be entered intact to upper intestinal tract. As the incorporation level of inulin was different, monitoring the fructan loss in IRWF‐supplemented formulations indicate a progressive fructan loss through processing in the range of 25.42%–43.01% w/w. In other words, the highest and lowest fructan loss is found in F15 and F1, respectively, which indicate the enhanced prebiotic loss by decreasing its incorporation level and polymerization degree. The enhanced inulin loss by decreasing its polymerization degree is attributed to its higher susceptibility to be used by yeast and take part in Millard reaction. This finding is in accordance with (Mohammadi et al., 2021).

## 4 CONCLUSION

Inulin supplementation of wheat bread in the form of inulin reconstituted wheat flour (IRWF) seems to be applicable from nutritional perspective, especially at formulations containing long‐chain inulin type. However, despite gluten inclusion at formulations to conquer gluten dilution impacts, adverse impacts on technological characteristics are still restricting in industrially production of them especially long‐chain inulin‐containing ones which are attributed to their higher water absorption content, accelerated gluten network formation, and decreased starch gelatinization. However, long‐chain inulin incorporation significantly improves the nutritional characteristics of wheat considering resistant starch content, hydrolysis index, predicted Glycemic index, and fructan loss parameters. Consequently, whole bread‐making process as the type of fermentation, time: temperature ratio of fermentation process and increasing ratio of temperature through baking process should be optimized to formulate optimized formulation from both nutritional and technological perspective.

## CONFLICT OF INTEREST

None declared.

## ETHICS STATEMENT

The Ethical aspects of this study are evaluated and approved by the Institutional Review Board of Shahid Sadoughi University of Medical Sciences.

## Data Availability

The authors confirm the availability of all data supporting the findings within the article.
